# Hesperetin mitigates acrolein-induced apoptosis in lung cells *in vitro* and *in vivo*

**DOI:** 10.1080/13510002.2018.1535640

**Published:** 2018-10-16

**Authors:** Jung Hyun Park, Hyeong Jun Ku, Jeen-Woo Park

**Affiliations:** a School of Life Sciences and Biotechnology, BK21 Plus KNU Creative BioResearch Group, Kyungpook National University, Taegu, Korea; b Department of Food and Biotechnology, Korea University, Sejong, Korea

**Keywords:** Acrolein, hesperetin, lung, apoptosis, reactive oxygen species

## Abstract

**Objectives:** A number of studies have suggested that acrolein-induced lung injury and pulmonary diseases are associated with the depletion of antioxidants and the production of reactive oxygen species. Therefore, compounds that scavenge reactive oxygen species may exert protective effects against acrolein-induced apoptosis. Because hesperetin, a natural flavonoid, has been reported to have an antioxidant activity, we investigated the effect of hesperitin against acrolein-induced apoptosis of lung cells.

**Methods:** We evaluated the protective role of hesperetin in acrolein-induced lung injury using Lewis lung carcinoma (LLC) cells and mice.

**Results:** Upon exposure of LLC cells and mice to acrolein, hesperetin ameliorated the lung inbjury through attenuation of oxidative stress.

**Conclusion:** In the present report, we demonstrate that hesperetin exhibits a protective effect against acrolein-induced apoptosis of lung cells in both *in vitro* and *in vivo* models. Our study provides a useful model to investigate the potential application of hesperetin for the prevention of lung diseases associated with acrolein toxicity.

## Introduction

Acrolein is a respiratory irritant that can be generated in cigarette smoke, as well as upon incomplete combustion of plastic materials and during cooking [1]. It plays a role in pulmonary inflammation and lung injury [2]. The inhalation of acrolein leads to acute lung injury, which is followed by the disruption of the alveolar-capillary barrier integrity, as well as by the development of pulmonary edema and chronic obstructive pulmonary disease [3,4]. It has been indicated that acrolein induces the production of reactive oxygen species (ROS) and the release of proinflammatory cytokines and this eventually leads to cellular apoptosis [5,6]. Acrolein, an α,β-unsaturated aldehyde, contains a highly reactive carbonyl group [7]. Similar to other reactive carbonyl species, acrolein is highly reactive with cellular nucleophiles such as proteins, DNA, and RNA. Acrolein readily targets and reacts with sulfhydryl or thiol groups and consequently depletes reduced glutathione (GSH), thioredoxin, and glutaredoxin, which are important antioxidants, therefore, there is increased oxidative stress [8–10].

Flavonoids are the most abundant polyphenols and they have various pharmacological properties [11]. It has been reported that flavonoids and members of their subclasses, including flavones, flavanones, flavonols, catechins, anthocyanidins, and isoflavones, act as antioxidants by scavenging free radicals [12]. Hesperetin (3′,5,7-trihydroxy-4-methoxyflavanone), a member of the flavanone subclass of flavonoids, is abundant in oranges and grapefruits, tomatoes and cherries [13]. Several studies have reported that hesperetin shows anti-inflammatory, antioxidant, anticarcinogenic, and neuroprotective effects [14,15]. Hesperetin has multiple OH groups which confer the greater antioxidant potency than is possessed by other flavanones [16,17]. Based on these previous studies, we hypothesized that pretreatment with hesperetin might have a protective effect on acrolein-induced lung injury via the antioxidant activity of the flavonoid.

In the present study, we demonstrated that hesperetin exhibited protective effects against acrolein-induced apoptosis of lung cells in both *in vitro* and *in vivo* models. Our study provides a useful model to investigate the potential application of hesperetin for the prevention of lung diseases associated with acrolein toxicity.

## Materials and methods

### Materials

β-NADP^+^, acrolein, hesperetin, rhodamine 123 and xylenol orange were purchased from Sigma Chemical Co. (St. Louis, MO, USA). 7-Amino-4-chloromethylcoumarin (CMAC) was purchased from Invitrogen (Waltham, MA, USA). Antibodies were purchased as follows: β-actin and cleaved caspase 3 (Santa Cruz Biotechnology, Santa Cruz, CA, USA); cytochrome C, phospho-p38 (p-p38), phospho-JNK (p-JNK), cleaved PARP, phospho-p53 (p-p53), and horseradish peroxidase (HRP)-conjugated secondary antibodies (Cell Signaling, Beverly, MA, USA); 4-hydroxynonenal (4-HNE) (Abcam, Cambridge, UK); peroxiredoxin-SO_3_ (Prx-SO_3_) (Abfrontier, Seoul, Korea); 8-OH-dG (Millipore, Billerica, MA, USA) antibodies.

### Cell culture

The Lewis lung carcinoma (LLC) cell line was purchased from the Japanese Collection of Research Bioresources Cell Bank (Osaka, Japan) and cultured in DMEM supplemented with 10% FBS and 1% penicillin/streptomycin. Cells were maintained at 37°C in a humidified CO_2_ chamber. At approximately 70% confluence, cells were preincubated with 30 μM hesperetin for 1 h and then incubated with acrolein for 1 h. An MTT assay was used to determine the cell viability.

### Animal protocol

Experiments were performed using 8-week-old male C57BL/6 mice. The animals were housed in a temperature- and humidity-controlled cage under a 12-h light–dark cycle and had free access to a standard diet and water. All animal experiments were performed in accordance with the protocols approved by the Institutional Animal Care and Use Committee of Kyungpook National University. Eight-week-old C57BL/6 mice, weighing approximately 21–24 g, were used for the experiments and segregated into four groups, with 3–6 mice per group (+vehicle, +hesperetin, +acrolein, and + hesperetin/acrolein). The mice were subjected to acute acrolein inhalation (10 ppm for 12 h) in a closed inhalation chamber in accordance with the previously published method [1], wherein hesperetin (60 mg/kg) was administered to mice intraperitoneally 1 h prior to acrolein exposure. After the treatment with acrolein, the mice were sacrificed, and the extracted lungs were stored in a deep freezer until the next experiments.

### Immunoblotting

Lung tissues from mice or LLC cells were homogenized with a dounce homogenizer in lysis buffer at 4°C. The homogenates were centrifuged, and the supernatants were transferred to different tubes, followed by measuring the protein concentrations. Samples (30 μg of protein) were subjected to 8-12% polyacrylamide gel electrophoresis. After electrophoresis, proteins were transferred onto nitrocellulose membranes, which were incubated with primary antibodies, followed by incubation with HRP-labeled anti-rabbit IgG. Immunoreactive bands were visualized using an enhanced chemiluminescence kit (Amersham Pharmacia Biotech, Buckinghamshire, UK) and exposure to an X-ray film.

### Immunofluorescence

Freshly prepared lung tissues, isolated from mice after acrolein treatment, were fixed in 4% formalin. The fixed lung tissues were washed with PBS with gentle agitation to remove excess formalin and then embedded in paraffin. The paraffin-embedded lung tissue was sliced at 5-μm thickness. For immunohistochemical analysis, slides were incubated with 3% BSA to block non-specific binding sites and then stained with appropriate primary antibodies at 4°C overnight. After washing three times with PBS, the slides were stained with Alexa Fluor-conjugated secondary antibodies in a box with dimmed light. The prepared slides were treated with a mounting medium containing an antiphotobleaching agent and sealed with a nail polish. The fluorochrome-labeled tissue was visualized under a confocal microscope, and a photograph was taken using a computer-based microscope imaging system (Carl Zeiss, Oberkochen, Germany).

### Histology

For H&E staining, a glass slide with attached tissue was stained with Hematoxylin Gill No. 3 for nuclear staining, a blueing solution for differentiation of stained nuclei, and eosin Y to distinguish the cytoplasm. All staining procedures were performed at room temperature with gentle agitation. The stained glass slide was dehydrated with graded ethanol, from 70% to 100%, and twice with xylene. An Elastic stain kit (HT25A-1KT), purchased from Sigma-Aldrich, was used to stain collagen fibers for the detection of fibrosis according to the manufacturer’s instruction. After finishing the staining procedure, the slides were sealed with a nail polish, and gaps were filled with a mounting medium. The sealed slides were left in a dark chamber for the solidification of the sealers, and then photographs were taken under light microscopy.

### Measurement of intracellular oxidation

The level of intracellular peroxides in LLC cells was evaluated by confocal microscopy with the oxidant-sensitive probe DCFH-DA. Mitochondrial membrane permeability transition (MPT) was measured by the incorporation of rhodamine 123 dye into the mitochondria. The intracellular GSH level was determined using a GSH-sensitive fluorescence dye CMAC [18]. Protein oxidation in LLC cells was assessed by immunoblot analysis using anti-Prx-SO_3_ antibody. The level of intracellular hydrogen peroxide was measured using the ferric-sensitive dye xylenol orange, which is based on the oxidizability of hydrogen peroxide to hydrogen peroxide to convert ferrous ions into ferric ions [19]. Lung tissue was homogenized, and the supernatants were collected by centrifugation. A total of 50 μg of protein was mixed with reaction buffer (25 mM ferrous ammonium sulfate, 1 M sorbitol, 0.25 M H_2_SO_4_, 1 mM xylenol orange). The mixture was incubated in the dark for 30 min at room temperature. The absorbance of xylenol orange, with its color changed by oxidized ferrous ions, was measured using a spectrophotometer at 560 nm. Levels of protein oxidation, lipid peroxidation, and DNA oxidation in lung tissues were determined by immunofluorescence using anti-Prx-SO_3_, anti-HNE and anti-8-OH-dG antibodies, respectively.

### Statistical analysis

All experiments were repeated three times, and data are presented as the mean ± SD. Data were analyzed by one-way ANOVA, followed by Student’s *t-*test, and *p *< .05 was considered statistically significant.

## Results

**Figure 1.**
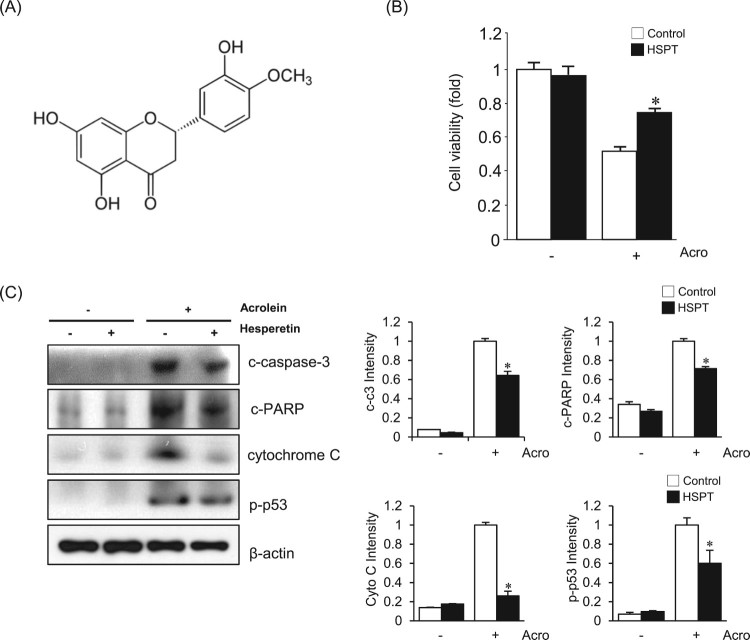
Effects of hesperetin on acrolein-induced toxicity to LLC cells. (A) The structure of hesperetin. (B) The viability of untreated (control) LLC cells and cells treated with 30 μM hesperetin (HSPT) upon exposure to 30 μM acrolein for 1 h was evaluated using an MTT assay. (C) Immunoblot analysis of apoptosis-related proteins. Cell extracts were electrophoresed on 10–15% SDS-polyacrylamide gels, transferred to nitrocellulose membranes, and immunoblotted with antibodies against cleaved caspase-3 (c-caspase-3), cleaved PARP (c-PARP), cytochrome C, and p-p53. β-Actin was used as an internal control. The protein levels were normalized to the actin levels to analyze the immunoblotting data. Data are presented as the mean ± SD of four independent experiments. *p < .05 versus cells exposed to acrolein. Acro, acrolein.

In this study, we first examined the effect of hesperetin (Figure 1(A)) on the toxicity of acrolein to LLC cells. To evaluate whether hesperetin has a protective effect, LLC cells were pretreated with 0–50 μM hesperetin for 1 h before incubation with acrolein. The range of acrolein concentrations was determined in preliminary experiments, and 30 μM acrolein was selected as an optimal concentration to mimic acute lung injury. After 1 h exposure to acrolein, cell viability was decreased to ∼50%. In contrast, more than 75% of cell viability was observed in the presence of 30 μM hesperetin (Figure 1(B)). To elucidate the mechanism of the effects of hesperetin against the acrolein-induced LLC cell death, cellular markers of apoptosis were measured. When LLC cells were pretreated with 30 μM hesperetin for 1 h before exposure to acrolein, the increased expression levels of apoptosis marker proteins, such as cleaved caspase-3, cleaved PARP, cytochrome C, and p-p53, were significantly attenuated (Figure 1(C)).

**Figure 2.**
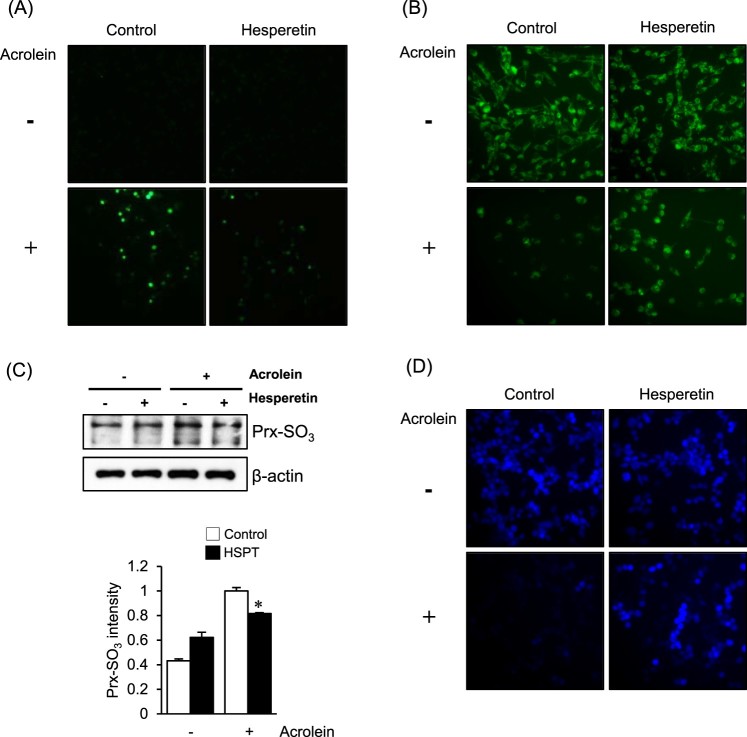
Effects of hesperetin on the cellular redox status and oxidative damage to LLC cells exposed to acrolein. (a) LLC cells were stained with DCFH-DA for 30 min, and DCF fluorescence was measured by fluorescence microscopy. (B) Mitochondrial membrane potential of LLC cells was measured by incorporation of the rhodamine 123 dye into mitochondria. (C) Immunoblot analysis of Prx-SO_3_ levels in LLC cell lysates. β-Actin was used as an internal control. The protein levels were normalized to the actin levels to analyze the immunoblotting data. Data are presented as the mean ± SD of four independent experiments. *p < .05 versus cells exposed to acrolein. (D) Fluorescence images of CMAC-loaded cells were acquired under a microscope to evaluate cellular GSH levels. HSPT, hesperetin.

As shown in Figure 2(A), an increase in DCF fluorescence, which reflects an elevated intracellular peroxide level, was observed in LLC cells when they were exposed to acrolein. The increase in fluorescence was significantly reduced in cells pretreated with 30 μM hesperetin for 1 h. Increased oxidative stress is accompanied by the disruption of the mitochondrial membrane potential; therefore, we evaluated the mitochondrial membrane potential with rhodamine 123 fluorescent dye. As shown in Figure 2(B), marked attenuation of fluorescence was observed in acrolein-treated LLC cells; however, hesperetin pretreatment significantly restored the level of fluorescence. Increased levels of oxidized peroxiredoxin (Prx-SO_3_), a marker for oxidative damage of the antioxidant enzyme Prx [20], were also found in LLC cells exposed to acrolein alone compared with those in cells pretreated with hesperetin before acrolein exposure (Figure 2(C)). Using the GSH-sensitive fluorescent dye CMAC, we found that the GSH levels in hesperetin-pretreated LLC cells exposed to acrolein were significantly recovered compared with those in acrolein-treated cells (Figure 2(D)).

**Figure 3.**
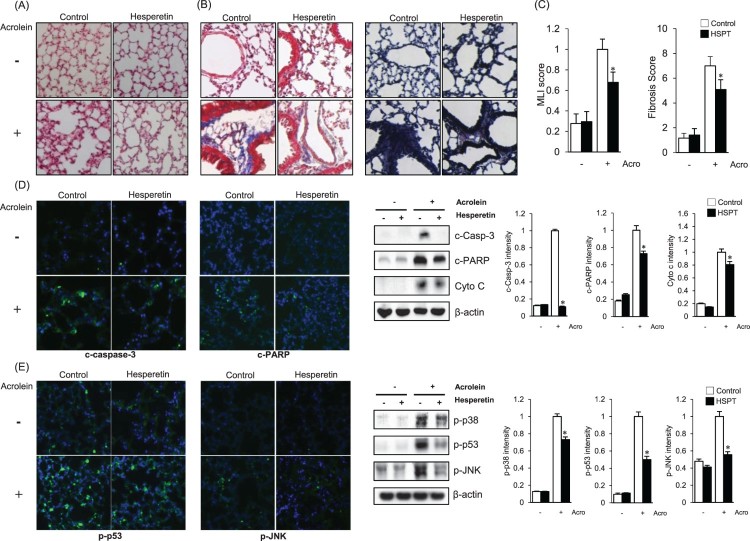
Effects of hesperetin on acrolein-induced lung damage in mice. Mice were exposed to filtered air or acrolein (10 ppm) for 12 h. Hesperetin (HSPT; 60 mg/kg) or PBS (control) was intraperitoneally administered to mice 1 h prior to acrolein exposure. (A) H&E-stained sections of lung tissues after acrolein exposure. (B) Mason trichrome staining and elastic staining of sections of lung tissues after acrolein exposure. (C) The Murray lung injury (MLI) score and fibrosis score were calculated based on the results of (A,B). Data are presented as the mean ± SD (*n* = 3–6 mice per group). *p < .05 versus acrolein-treated mice. (D) Immunofluorescence and immunoblot data comparing the levels of apoptotic marker proteins in lung tissue extracts from acrolein-treated mice. β-Actin was used as an internal control. (E) Immunofluorescence and immunoblot data comparing the levels of p-p38, p-p53, and p-JNK in lung tissue extracts from acrolein-treated mice. β-Actin was used as an internal control. In (D) and (E), the protein levels were normalized to the β-actin levels to analyze the immunoblotting data. Data are presented as the mean ± SD (*n* = 3–6 mice per group). *p < .05 versus acrolein-treated mice. Acro, acrolein.

To investigate the protective effects of hesperetin on acrolein-induced lung damage *in vivo*, hesperetin (60 mg/kg) was intraperitoneally administered to mice 1 h prior to acrolein exposure. The mice were exposed to 10 ppm acrolein or filtered air for 2 h. Because acrolein is known to modify the protein structure [21], we stained lung tissue with hematoxylin and eosin Y to observe the change in its structure. As shown in Figure 3(A), severely destructed and irregularly patterned alveoli were observed in the acrolein-treated group, but these histological characteristics were markedly ameliorated by hesperetin. Masson’s trichrome staining and elastic staining were performed to evaluate whether fibrosis, a well-known diagnostic marker of lung injury, occurred in the lung upon acrolein exposure. Pulmonary fibrosis is known to induce irreversible structure modification and is regarded as one of the major causes of death through the initiation of apoptosis [22,23]. In Figure 3(B), stained fibers are represented by the blue color in the left panel and black color in the right panel. The degree of lung injury was calculated as the fibrosis score and Murray lung injury (MLI) score using ImageJ and presented as a graph. Significant protective effects of hesperetin, able to reduce the susceptibility of mice to acrolein-induced lung injury, were documented as a significant attenuation of the lung injury score (Figure 3(C)). To gain further insight into the effect of hesperetin against lung damage in acrolein-treated mice, we studied the effect of hesperetin on the process of apoptosis. As shown in Figure 3(D), a higher expression of cleaved caspase-3 and cleaved PARP, which represent the apoptotic index, was observed in the lung tissues of acrolein-treated mice. However, these markers of apoptosis were markedly attenuated by pretreatment with hesperetin. Western blot analysis of cleaved caspase-3, cleaved PARP, and cytochrome C, a marker of mitochondrial apoptosis, further confirmed the data of immunofluorescence analysis. Immunofluorescence analysis reveals that the levels of p-p53 and p-JNK increased in the lung tissues of acrolein-treated mice, however, these proteins were markedly reduced by hesperetin (Figure 3(E)). Western blot analysis confirms that the expression levels of the activated forms of p38 (p-p38), p53 (p-p53), and JNK (p-JNK) were attenuated in acrolein-administered hesperetin-pretreated mice compared to those in the mice treated with acrolein alone (Figure 3(E)).

**Figure 4.**
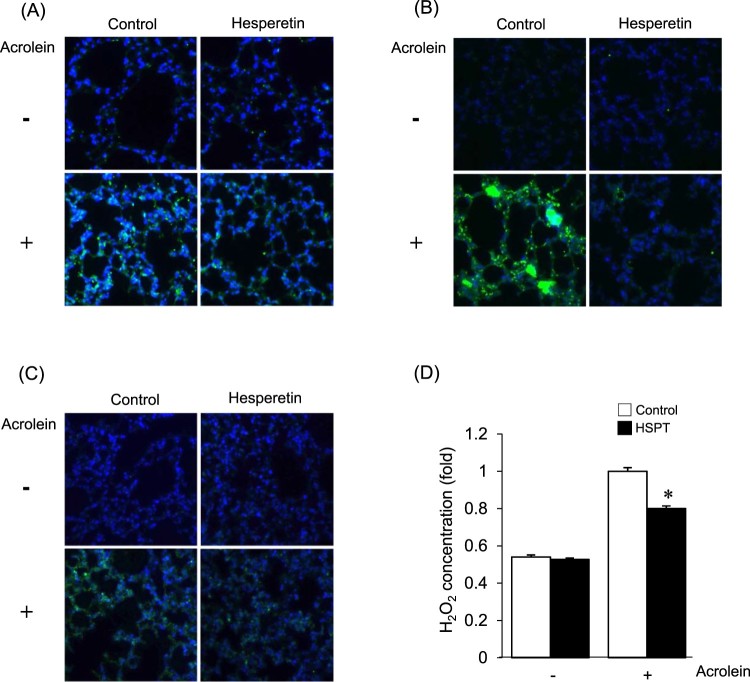
Effects of hesperetin on the modulation of the redox status by acrolein in mice. Hesperetin (HSPT) or PBS (control) was administered to mice prior to acrolein exposure. Immunofluorescence analysis of the levels of Prx-SO_3_ (A), HNE protein adducts (B), and 8-OH-dG (C) in lung tissues of mice. (D) Intracellular hydrogen peroxide in lung tissue extracts was measured using xylenol orange. The level of hydrogen peroxide of lung tissue from acrolein-exposed control mice was expressed as 1. Data are presented as the mean ± SD (*n* = 3–6 mice per group). *p < .05 versus acrolein-treated mice.

To confirm that increased oxidative stress is responsible for the acrolein-induced damage, the effect of hesperetin on acrolein toxicity was evaluated *in vivo*. Upon exposure to acrolein, the level of Prx-SO_3_ in lung tissue was significantly lower in hesperetin-pretreated mice than in untreated mice (Figure 4(A)). Lipid peroxidation was increased in the lung tissue of acrolein-treated mice and was significantly suppressed by pretreatment with hesperetin (Figure 4(B)). After acrolein treatment, the 8-OH-dG level in endogenous DNA significantly increased and hesperetin suppressed 8-OH-dG formation (Figure 4(C)). To determine whether the differences in acrolein-induced lung cell death between hesperetin-treated and untreated mice were associated with ROS formation, the levels of intracellular hydrogen peroxide in lung tissue were measured using xylenol orange. As depicted in Figure 4(D), a significantly higher level of intercellular hydrogen peroxide was observed in the lung tissue of the mice treated with acrolein alone compared with that in hesperetin-pretreated mice.

## Discussion

Our observations are consistent with the hypothesis that the apoptotic cell death observed upon exposure to acrolein is associated with ROS formation and oxidative stress. Acrolein is known to induce oxidative stress through interactions with other molecules, owing to its high reactivity and unstable molecular structure [24,25]. GSH is a well-known antioxidant, which is usually present as the most abundant low-molecular-mass thiol in most organisms. It has various functions in the defense against oxidative stress and xenobiotic toxicity [26]. The cellular levels of GSH are closely associated with many cell death-inducing effects of acrolein [27,28]. The GSH levels in hesperetin-pretreated LLC cells exposed to acrolein were significantly recovered compared with those in acrolein-treated cells. In addition, the pretreatment of hesperetin significantly improved redox status and inhibited the apoptotic pathway in LLC cells. These results strengthen the conclusion that hesperetin protected cells from acrolein-induced apoptosis by decreasing the steady-state levels of intracellular oxidants and by preventing GSH depletion.

To investigate the protective effects of hesperetin on acrolein-induced lung damage *in vivo*, hesperetin was treated to mice prior to acrolein exposure. The diagnostic markers of lung injury induced by acrolein, such as the altered lung tissue structure and pulmonary fibrosis, were markedly ameliorated by hesperetin. In addition, the severity of acrolein-induced lung damage can be reduced by hesperetin, which influences the level of apoptosis. To further elucidate the effect of hesperetin on the proapoptotic signaling pathway, activation of p53, p38 and JNK was examined. ROS promote apoptosis by stimulating proapoptotic signaling molecules such as JNK and p38 MAPK [29]. It is speculated that ROS might be the main regulator of p53 activation through the JNK pathway and to activate p38 in apoptotic cell death [30]. The expression levels of the activated forms of p38, p53, and JNK were attenuated in acrolein-administered hesperetin-pretreated mice compared to those in the mice treated with acrolein alone, indicating that the p38-JNK and p53 pathway contribute to the acrolein-induced cell death.

The present study demonstrated that hesperetin protected LLC cells and lung tissue of mice against acrolein-induced toxicity through its antioxidative effects. Our study provides a useful model to investigate the potential application of hesperetin for the treatment or prevention of acrolein toxicity.
